# Estimating the Instantaneous Asymptomatic Proportion With a Simple Approach: Exemplified With the Publicly Available COVID-19 Surveillance Data in Hong Kong

**DOI:** 10.3389/fpubh.2021.604455

**Published:** 2021-05-03

**Authors:** Chunyu Li, Shi Zhao, Biao Tang, Yuchen Zhu, Jinjun Ran, Xiujun Li, Daihai He

**Affiliations:** ^1^Department of Biostatistics, School of Public Health, Cheeloo College of Medicine, Shandong University, Jinan, China; ^2^JC School of Public Health and Primary Care, Chinese University of Hong Kong, Hong Kong, China; ^3^Chinese University of Hong Kong (CUHK) Shenzhen Research Institute, Shenzhen, China; ^4^School of Mathematics and Statistics, Xi'an Jiaotong University, Xi'an, China; ^5^Laboratory for Industrial and Applied Mathematics, Department of Mathematics and Statistics, York University, Toronto, ON, Canada; ^6^School of Public Health, Li Ka Shing Faculty of Medicine, University of Hong Kong, Hong Kong, China; ^7^Department of Applied Mathematics, Hong Kong Polytechnic University, Hong Kong, China

**Keywords:** COVID-19, likelihood-based framework, instantaneous asymptomatic proportion, Hong Kong, epidemic

## Abstract

**Background:** The asymptomatic proportion is a critical epidemiological characteristic that modulates the pandemic potential of emerging respiratory virus, which may vary depending on the nature of the disease source, population characteristics, source–host interaction, and environmental factors.

**Methods:** We developed a simple likelihood-based framework to estimate the instantaneous asymptomatic proportion of infectious diseases. Taking the COVID-19 epidemics in Hong Kong as a case study, we applied the estimation framework to estimate the reported asymptomatic proportion (rAP) using the publicly available surveillance data. We divided the time series of daily cases into four stages of epidemics in Hong Kong by examining the persistency of the epidemic and compared the rAPs of imported cases and local cases at different stages.

**Results:** As of July 31, 2020, there were two intermittent epidemics in Hong Kong. The first one was dominated by imported cases, accounting for 63.2% of the total cases, and the second one was dominated by local cases, accounting for 86.5% of the total cases. The rAP was estimated at 23.1% (95% CI: 10.8–39.7%) from January 23 to July 31, and the rAPs were estimated at 22.6% (95% CI: 11.1–38.9%) among local cases and 38.7% (95% CI: 9.0–72.0%) among imported cases. Our results showed that the rAPs of local cases were not significantly different between the two epidemics, but increased gradually during the first epidemic period. In contrast, the rAPs of imported cases in the latter epidemic period were significantly higher than that in the previous epidemic period.

**Conclusion:** Hong Kong has a high rAP of imported COVID-19 cases and should continue to strengthen the detection and isolation of imported individuals to prevent the resurgence of the disease.

## Introduction

An atypical pneumonia case in early December 2019 caught the attention of medical institutions and was later confirmed to be novel coronavirus disease 2019 (COVID-19) caused by the severe acute respiratory syndrome coronavirus-2 (SARS-CoV-2) (1, 2). Since early December 2019, the disease has spread rapidly around the world, with many countries and regions reporting an exponential increase in confirmed cases. In the face of tensions all over the world, the World Health Organization announced that the COVID-19 outbreak was considered as a Public Health Emergency of International Concern since January 31, and eventually classified it as a pandemic on March 11, 2020 (3). As of August 16, 2020, 216 countries and territories had reported more than 21 million confirmed cases, including 760,000 deaths (4). While these numbers are horrifying, they are only a fraction of those infected.

Most of the COVID-19 infections appear to have two outcomes, some become severely ill or even fatal (symptomatic infections), while others show no symptom (asymptomatic infections) (5). In other words, asymptomatic infected individuals are defined as those who have positive RT-PCR testing outcome without any symptom. The asymptomatic COVID-19 infections have been frequently reported since January 2020 and take a large ratio of the total COVID-19 cases (6–8). Several studies showed that the viral load of asymptomatic individuals is similar to that of symptomatic cases, which suggested that asymptomatic individuals can also promote the spread of the disease (9, 10). In parallel, He et al. (11) showed that infectiveness of asymptomatic cases was 25% of that related to the symptomatic ones. Moreover, Day et al. (12) showed that the majority (from 50 to 75%) of people infected with COVID-19 were asymptomatic, but represented “*a formidable source*” of contagion. On the other hand, a previous study reported that asymptomatic individuals can still transmit the pathogen even 14 days later after they become infectious (5).

Based on these evidences mentioned above, we can see that it is fundamental to estimate the proportion of asymptomatic cases, further evaluate the impact of it on the disease burden and the effectiveness of the control interventions, and finally provide the decision-making basis in controlling the spread of the diseases (13–17). At present, many studies have estimated the asymptomatic proportion of total COVID-19-infected cases at different sites by observational studies or mathematical models (18–25). These estimated proportions were raw rates or assumed to remain constant over time. However, asymptomatic proportion may vary depending on the nature of the disease source, population characteristics (e.g., age structure, sex, health status, immune status, and genetic characteristics), pathogen–host interaction, and environmental factors. At the same time, in several countries or regions, the COVID-19 epidemics resurge and have a second wave of peak after a brief respite. It remains unknown whether the instantaneous asymptomatic proportion will change during this process (6).

The main purpose of this study is to develop a simple likelihood-based but generalized framework to estimate the instantaneous asymptomatic proportions for uncovering the features of COVID-19, thereby providing insights into understanding the spread of epidemics. Taking the epidemics in Hong Kong as a case study, we demonstrate the estimation framework by using the publicly available COVID-19 surveillance data.

## Methods

### Estimation Framework

We denote the time interval between symptoms onset (if symptomatic) and being confirmed as τ, and let *f* (τ) be the probability distribution function (PDF) of τ. That is, if one case is reported on date *t* who becomes symptomatic eventually, the value of *f* (τ) is considered as the relative likelihood of symptoms onset on date (*t* + τ).

We assume that all symptomatic cases will be confirmed (most likely in Hong Kong), while confirmed cases can be symptomatic, pre-symptomatic, or asymptomatic at the time of reporting. Thus, the term τ need not necessarily be positive; i.e., negative values are also possible theoretically. Hence, we consider all the confirmed cases as the “pool” of symptomatic cases, and we model this candidate pool as a time-varying function denoted by *Φ*(*t*) on date *t*. On date *t*, the *i*th case, who is reported on date υ_*i*_, contributes *f* (*τ* = *υ*_*i*_ – *t*) to *Φ*(*t*). For the contribution from all reported cases, *Φ*(*t*) is summated as in Equation (1).

(1) $$\begin{array}{l} \Phi \left(t\right)=\underset{i}{\sum }f\left(\tau =\upsilon_{i}-t\right) \end{array}$$

Hence, the reported asymptomatic proportion (rAP), i.e., the asymptomatic proportion among reported cases, on date *t* is calculated by rAP_*t*_ = 1 − *α*_*t*_/*Φ*_*t*_. Here, α_*t*_ is the observed number of cases with symptoms onset on date *t*, and *Φ*_*t*_ is the discretized *Φ*(*t*) on date *t*.

Given the infection time of one case (as condition), the onset time of this case is conditionally independent from each other case. Thus, to construct the likelihood profile, we model α_*t*_ as a binomial process with sizes at *Φ*_*t*_ (rounding to the closest integer) and successful probabilities at rAP_*t*_ to be estimated. As such, by fitting to the daily number of symptomatic cases time series, the rAP_*t*_ can be estimated by using the maximum likelihood estimation approach. The 95% confidence intervals (95% CI) of rAP_*t*_s are calculated by using the profile likelihood estimation framework with a cutoff threshold determined by a Chi-square quantile (26), as well as previously adopted in (27–32).

### COVID-19 Surveillance Data in Hong Kong

For demonstration, we used the publicly available COVID-19 surveillance data from January 23 to August 8, 2020 in Hong Kong as an example to construct the instantaneous rAPts series. The daily reported number of COVID-19 cases and date of onset were collected from https://www.coronavirus.gov.hk/eng/index.html. A laboratory-confirmed case was defined if the patient had a positive test of SARS-CoV-2 virus by the real-time reverse-transcription-polymerase-chain-reaction (RT-PCR) assay or high-throughput sequencing of nasal and pharyngeal swab specimens (33). Only laboratory-confirmed cases were included in this study.

We divided the time series of daily cases in Hong Kong into different stages of the epidemic by examining the persistency of the epidemic. In this study, we considered the criterion that the epidemic persists with the daily number of cases larger than 5 for three consecutive days, but does not persist otherwise. Following this criterion, we have the following four stages of the epidemic, and they included:

- stage (**I**): from January 23 to March 7, with sporadic cases,
- stage (**II**): from March 8 to April 3, with an epidemic peak,
- stage (**III**): from April 4 to June 30, with sporadic cases, and
- stage (**IV**): from July 1 to July 31, with another epidemic peak.

Based on the estimated instantaneous asymptomatic proportion, we summarized the pooled asymptomatic proportions during the four different stages. To avoid the estimation inaccuracy due to reporting delay, we excluded the data from August 1 to August 8, 2020, and conducted the estimation using the remaining dataset.

To set up the initial conditions of the model framework in Equation (1), we initialized the PDF *f* (·) by a gamma distribution. Although τ can be negative theoretically, the situation when the report is prior to the symptoms onset rarely occurs in Hong Kong (only 1 out of a total of 3,067 symptomatic cases). Thus, for simplification, we model *f* (·) as PDF defined all positive values, which will not affect our main conclusions. We fitted gamma distribution *f* (·) to the observed time intervals between symptoms onset and being reported, and the parameters of *f* (·) are estimated by using the maximum likelihood estimation. We estimated the mean at 6.0, 7.2, or 5.7 days, and standard deviation (SD) at 4.3, 6.6, or 3.4 days for all, imported, or local COVID-19 cases, respectively. These estimates are implemented to set up *f* (·) in Equation (1) for further rAP estimation.

## Results

**Figure 1:**
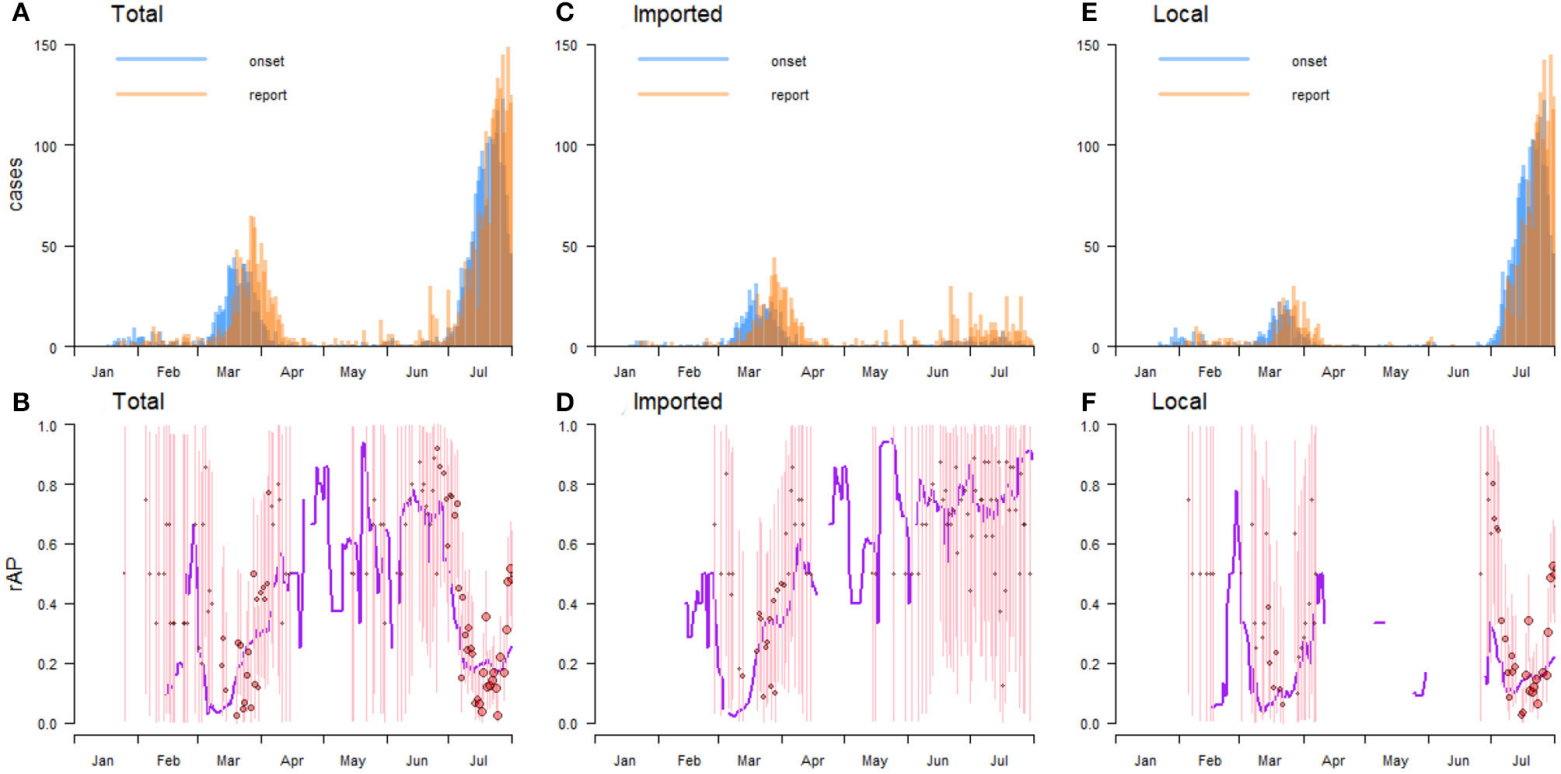
The daily number of total **(A)**, imported **(C)**, and local **(E)** COVID-19 confirmed cases in Hong Kong. The daily reported asymptomatic proportions (rAPs) among the total **(B)**, imported **(D)**, and local **(F)** cases. In panels **(B,D,F)**, the red circles represent the daily rAP, where the circle size represents the sample size, the pink vertical bar represents the 95% confidence interval (95% CI), and the purple curve represents the crude rAP.

By July 31, a total of 3,272 cases were reported in Hong Kong, of which 67.2% were locals and 32.8% were imported, respectively. In particular, the first two cases were reported on January 23, 2020, and both were imported. In stage (**I**), all cases were imported in the first week, after which the local transmission emerges and the local cases gradually dominate the total cases, with local cases accounting for 70.9% of the total cases (78 out of 110 cases). In stage (**II**), the daily number of COVID-19 confirmed cases increased rapidly and reached the peak of 44 new cases on March 19. After that, the daily number of cases gradually declined till April 4, 2020, when it dropped to below 5. A total of 753 confirmed cases were reported in stage (**II**), most of them were imported, accounting for 63.2% of the total cases. In stage (**III**), only sporadic new cases emerged every day. A total of 343 cases were reported within about 3 months, of which the vast majority (83.7%) were imported. From July 1, 2020, the epidemics in Hong Kong entered its stage (**IV**), during which the number of daily cases increased rapidly again with a peak of 122 cases on July 7, 2020, and then declined gradually. The epidemic intensity of stage (**IV**) was 2.7-fold, i.e., 2,066 vs. 753 total number of cases, higher than that of stage (**II**). Different from the previous stages, local cases dominated the epidemic in stage (**IV**), accounting for 86.5% of the total cases (1,787 out of 2,066 cases), while only 13.5% of the cases were imported (Table 1 and Figure 1).

**Table 1:** Summary of cases and the estimated asymptomatic proportions in the reported imported and local COVID-19 confirmed cases (rAP) at different stages of the epidemic in Hong Kong.

**Stage**	**Period**	**# of cases**			**Daily cases**	**rAP**	
		**Total**	**Imported**	**Local**		**Imported**	**Local**
(**I**)	Jan 23–Mar 7	110	32 (29.1%)	78 (70.9%)	2.4	51.9% (5.7–94.6%)	54.6% (2.3–99.1%)
(**II**)	Mar 8–Apr 3	753	476 (63.2%)	277 (36.8%)	27.9	27.3% (6.6–60.1%)	22.4% (2.6–64.1%)
(**III**)	Apr 4–Jun 30	343	287 (83.7%)	56 (16.3%)	3.9	68.0% (15.8–98.5%)	59.4% (9.9–98.8%)
(**IV**)	Jul 1–Jul 31	2,066	279 (13.5%)	1,787 (86.5%)	66.6	56.4% (13.2–91.6%)	22.2% (11.9–36.2%)
Pooled est.	Jan 23–Jul 31	3,272	1,074 (32.8%)	2,198 (67.2%)	17.1	38.7% (9.0–72.0%)	22.6% (11.1–38.9%)

*The estimates are showed in “point estimate (95% CI)” format*.

In Hong Kong, the pooled rAP was estimated at 23.1% (95% CI: 10.8–39.7%) from January 23 to July 31, and the rAPs were estimated at 22.6% (95% CI: 11.1–38.9%) among local cases and 38.7% (95% CI: 9.0–72.0%) among imported cases. In stage (**I**), the rAPs fluctuated considerably, especially in local cases. After entering stage (**II**), the rAPs were low at the beginning, but increased gradually as the epidemic progressed. At this stage, the increasing trends of rAPs of local and imported cases were similar, but the one for local cases was generally lower than that for imported cases. In stage (**III**), asymptomatic individuals were mainly imported, while the local cases were negligible. The rAPs were relative volatile in stage (**III**), similar to those in stage (**I**). In stage (**IV**), the rAPs of local cases were relatively stable, maintaining around 22%, while the rAPs of imported cases fluctuated greatly, reaching higher than 60% in about half of the time period (Table 1 and Figure 1).

## Discussion

We developed a simple likelihood-based framework to estimate the instantaneous asymptomatic proportion of infectious diseases and used the publicly available COVID-19 surveillance data in Hong Kong as an example for demonstration.

As an international metropolis, Hong Kong has a high population mobility. Imported cases account for a significant proportion of reported cases, particularly during the first epidemic. We found that the pooled rAP estimation of imported cases (38.7%) appears higher than that of local cases (22.6%). Several potential factors led to this result. Firstly, imported cases are mainly from the United Kingdom and the United States (34), and the asymptomatic rate reflects the comprehensive level of the importing countries. Some representative studies have shown that the asymptomatic infection rate is around 40% (35, 36), which is consistent with our estimated rAP at 38.7% (95% CI: 9.0–72.0) among imported cases. Secondly, imported cases are mainly returned from overseas study or tourism. They are mostly young people, with a large proportion aged from 15 to 24 (11, 34). However, elder people appear more likely to develop severe symptoms, as shown in the epidemic on the Diamond Princess cruise ship (24, 37). Thirdly, asymptomatic infections rarely seek medical advice and thus are less likely ascertained. However, during the outbreak, the Hong Kong government quarantined all arrivals, which can lead to more stringent testing and quarantine for imported than local individuals. As such, asymptomatic individuals in imported cases can be more fully captured.

The time series of the COVID-19 epidemic in Hong Kong included in our study was divided into four stages, of which stages (**II**) and (**IV**) were two discontinuous epidemic periods. The trend of rAPs, proportion of imported cases, and rAPs of imported cases changed greatly between the two epidemic periods. During an epidemic, the testing coverage and the level of contact tracing should be constantly increased, which results in a significant increase of the detection rate of asymptomatic infected individuals (38), that is, leads to a gradual increase in instantaneous asymptomatic rates of both imported cases and local cases. Most Hong Kong residents who studied or traveled abroad returned on or before the first epidemic period (34). With the development of the epidemic, public awareness may gradually increase. COVID-19 infected cases with symptoms will choose to travel less, hence less likely to import into Hong Kong. Meanwhile, the suspension of most airlines and shipping to Hong Kong with a strengthened quarantine rate and the spread of portable devices such as thermometers have also contributed to the less imported cases. These factors played important roles in reducing the proportion of imported cases and increasing the rAPs of imported cases.

There were sporadic daily cases in stages (**I**) and (**III**). At these two stages, a slight change in the number of asymptomatic infected individuals could cause a drastic fluctuation of the rAPs; consequently, the confidence intervals of estimation are relatively large.

The data-driven rAP depends highly on the precise ratio of asymptomatic infected individuals and symptomatic cases. We found that the reporting of asymptomatic individuals may have significantly influenced the scale of case data, as symptomatic cases are less likely to be incorrectly identified (or under-ascertainment rate to be relatively low). Therefore, on one hand, the under-ascertainment in asymptomatic individuals can result in an underestimation of the rAPs. On the other hand, if we assume the asymptomatic proportion ranging from 27.8 to 30.8% among clinically diagnosable COVID-19 cases as estimated in previous studies (19, 21), an average under-ascertainment rate of asymptomatic individuals in Hong Kong ranging from 22.0 to 32.5% is calculated backwardly.

For another aspect, asymptomatic COVID-19 cases may have important contributions to secondary infections (39). They can unknowingly spread the virus and are more likely to produce asymptomatic offspring, bringing severe battles for epidemic prevention and control (40). In this study, we proposed an analytical approach to estimate the instantaneous asymptomatic proportion of infectious diseases and, as a case study, to reveal the temporal patterns of COVID-19 transmission and spectrum. We believe that our study can bring an insight into understanding the transmission of COVID-19. It should be pointed out that our study still has several limitations. Firstly, our estimates rely on total and timely reporting of asymptomatic infected individuals. Alternatively, an overdispersion setting in the likelihood distribution can be incorporated to resolve inaccurate deterministic scenarios. Secondly, as a data-driven analysis, our estimates rely on the consistency of the statistical framework and reported COVID-19 case data.

## Data Availability Statement

Publicly available datasets were analyzed in this study. This data can be found here: https://www.chp.gov.hk/files/pdf/local_situation_covid19_en.pdf.

## Ethics Statement

The ethical approval and individual consents were exempted as the aggregated data used in this study are from public domain.

## Author Contributions

All authors conceived and conducted the research, wrote the draft, critically revised the manuscript, and approved the submission.

## Conflict of Interest

DH received support from an Alibaba (China)—Hong Kong Polytechnic University Collaborative Research project. The remaining authors declare that the research was conducted in the absence of any commercial or financial relationships that could be construed as a potential conflict of interest.

## References

[1] Chen N Zhou M Dong X Qu J Gong F Han Y. Epidemiological and clinical characteristics of 99 cases of 2019 novel coronavirus pneumonia in Wuhan, China: a descriptive study. Lancet. (2020) 395:507–13. 10.1016/S0140-6736(20)30211-7 32007143 PMC7135076

[2] Guan W Ni Z Hu Y Liang W Ou C He J. Clinical characteristics of coronavirus disease 2019 in China. N Engl J Med. (2020) 382:1708–20. 10.1056/NEJMoa2002032 32109013 PMC7092819

[3] Maier BF Brockmann D. Effective containment explains subexponential growth in recent confirmed COVID-19 cases in China. Science. (2020) 368:742–6. 10.1126/science.abb4557 32269067 PMC7164388

[4] WHO. Coronavirus Disease (COVID-19) Situation Report-209. (2020). Available online at: https://www.who.int/docs/default-source/coronaviruse/situation-reports/20200816-covid-19-sitrep-209.pdf?sfvrsn=5dde1ca2_2 (cited August 24, 2020).

[5] Oran DP Topol EJ. Prevalence of asymptomatic SARS-CoV-2 infection: A narrative review. Ann Intern Med. (2020) 173:362–7. 10.7326/M20-3012 32491919 PMC7281624

[6] Gangakhedkar GR Sundaram S Gangakhedkar MR Shilotri MP. hazardous postoperative outcomes of unexpected COVID-19 infected patients: a call for global consideration of sampling all asymptomatic patients before surgical treatment. World J Surg. (2020) 44:3192–3. 10.1007/s00268-020-05659-z 32591843 PMC7319489

[7] Bae SH Shin H Koo H-Y Lee SW Yang JM Yon DK. Asymptomatic transmission of SARS-CoV-2 on evacuation flight. Emerg Infect Dis. (2020) 26:2705–8. 10.3201/eid2611.203353 32822289 PMC7588520

[8] Ali M Shah STH Imran M Khan A. The role of asymptomatic class, quarantine and isolation in the transmission of COVID-19. J Biol Dyn. (2020) 14:389–408. 10.1080/17513758.2020.1773000 32498655

[9] Bai Y Yao L Wei T Tian F Jin DY Chen L. Presumed asymptomatic carrier transmission of COVID-19. JAMA. (2020) 323:1406–7. 10.1001/jama.2020.2565 32083643 PMC7042844

[10] Zou L Ruan F Huang M Liang L Huang H Hong Z. SARS-CoV-2 viral load in upper respiratory specimens of infected patients. N Engl J Med. (2020) 382:1177–9. 10.1056/NEJMc2001737 32074444 PMC7121626

[11] He D Zhao S Lin Q Zhuang Z Cao P Wang MH. The relative transmissibility of asymptomatic COVID-19 infections among close contacts. Int J Infect Dis. (2020) 94:145–7. 10.1016/j.ijid.2020.04.034 32315808 PMC7166025

[12] Day M. Covid-19: identifying and isolating asymptomatic people helped eliminate virus in Italian village. BMJ. (2020) 368:m1165. 10.1136/bmj.m1165 32205334

[13] He D Zhao S Lin Q Zhuang Z Cao P Wang MH. Risk for transportation of coronavirus disease from Wuhan to other cities in China. Emerg Infect Dis. (2020) 26:1049–52. 10.3201/eid2605.200146 32053479 PMC7181905

[14] Munster VJ Koopmans M Doremalen N van Riel D van Wit E de. A novel coronavirus emerging in China - Key questions for impact assessment. N Engl J Med. (2020) 382:692–4. 10.1056/NEJMp2000929 31978293

[15] Wu P Hao X Lau EHY Wong JY Leung KSM Wu JT. Real-time tentative assessment of the epidemiological characteristics of novel coronavirus infections in Wuhan, China, as at 22 January 2020. Eurosurveillance. (2020) 25:2000044. 10.2807/1560-7917.ES.2020.25.3.2000044 31992388 PMC6988272

[16] Chan JFW Yuan S Kok KH To KKW Chu H Yang J. A familial cluster of pneumonia associated with the 2019 novel coronavirus indicating person-to-person transmission: a study of a family cluster. Lancet. (2020) 395:514–23. 10.1016/S0140-6736(20)30154-9 31986261 PMC7159286

[17] Li R Pei S Chen B Song Y Zhang T Yang W. Substantial undocumented infection facilitates the rapid dissemination of novel coronavirus (SARS-CoV-2). Science. (2020) 368:489–93. 10.1126/science.abb3221 32179701 PMC7164387

[18] Hu Z Song C Xu C Jin G Chen Y Xu X. Clinical characteristics of 24 asymptomatic infections with COVID-19 screened among close contacts in Nanjing, China. Sci China Life Sci. (2020) 63:706–11. 10.1007/s11427-020-1661-4 32146694 PMC7088568

[19] Qiu H Wu J Hong L Luo Y Song Q Chen D. Clinical and epidemiological features of 36 children with coronavirus disease 2019 (COVID-19) in Zhejiang, China: an observational cohort study. Lancet Infect Dis. (2020) 20:689–96. 10.1016/S1473-3099(20)30198-5 32220650 PMC7158906

[20] Kimball A Hatfield KM Arons M James A Taylor J Spicer K. Asymptomatic and presymptomatic SARS-CoV-2 infections in residents of a long-term care skilled nursing facility - King County, Washington, March 2020. MMWR Morb Mortal Weekly Rep. (2020) 69:377–81. 10.15585/mmwr.mm6913e1 32240128 PMC7119514

[21] Nishiura H Kobayashi T Miyama T Suzuki A Jung S-M Hayashi K. Estimation of the asymptomatic ratio of novel coronavirus infections (COVID-19). Int J Infect Dis. (2020) 94:154–5. 10.1016/j.ijid.2020.03.020 32179137 PMC7270890

[22] Zhou X Li Y Li T Zhang W. Follow-up of asymptomatic patients with SARS-CoV-2 infection. Clin Microbiol Infect. (2020) 26:957–9. 10.1016/j.cmi.2020.03.024 32234453 PMC7271011

[23] Al-Tawfiq JA. Asymptomatic coronavirus infection: MERS-CoV and SARS-CoV-2 (COVID-19). Travel Med Infect Dis. (2020) 35:101608. 10.1016/j.tmaid.2020.101608 32114075 PMC7102602

[24] Mizumoto K Kagaya K Zarebski A Chowell G. Estimating the asymptomatic proportion of coronavirus disease 2019 (COVID-19) cases on board the Diamond Princess cruise ship, Yokohama, Japan, 2020. Eurosurveillance. (2020) 25:2000180. 10.2807/1560-7917.ES.2020.25.10.2000180 32183930 PMC7078829

[25] Quilty BJ Clifford S Flasche S Eggo RM. Effectiveness of airport screening at detecting travellers infected with novel coronavirus (2019-nCoV). Eurosurveillance. (2020) 25:2000080. 10.2807/1560-7917.ES.2020.25.5.2000080 32046816 PMC7014668

[26] Fan J Huang T. Profile likelihood inferences on semiparametric varying-coefficient partially linear models. Bernoulli. (2005) 11:1031–57. 10.3150/bj/1137421639

[27] Zhao S Lou Y Chiu APY He D. Modelling the skip-and-resurgence of Japanese encephalitis epidemics in Hong Kong. J Theor Biol. (2018) 454:1–10. 10.1016/j.jtbi.2018.05.017 29792875 PMC7094098

[28] Zhao S Stone L Gao D Musa SS Chong MKC He D. Imitation dynamics in the mitigation of the novel coronavirus disease (COVID-19) outbreak in Wuhan, China from 2019 to 2020. Ann Transl Med. (2020) 8:448. 10.21037/atm.2020.03.168 32395492 PMC7210122

[29] Zhao S Musa SS Lin Q Ran J Yang G Wang W. Estimating the unreported number of novel coronavirus (2019-nCoV) cases in China in the first half of January 2020: a data-driven modelling analysis of the early outbreak. J Clin Med. (2020) 9:388. 10.3390/jcm9020388 32024089 PMC7074332

[30] Zhao S. Estimating the time interval between transmission generations when negative values occur in the serial interval data: using COVID-19 as an example. Math Biosci Eng. (2020) 17:3512–9. 10.3934/mbe.2020198 32987541

[31] Wang K Zhao S Liao Y Zhao T Wang X Zhang X. Estimating the serial interval of the novel coronavirus disease (COVID-19) based on the public surveillance data in Shenzhen, China, from 19 January to 22 February 2020. Transboundary Emerg Dis. (2020) 67:2818–22. 10.1111/tbed.13647 32452648 PMC7283843

[32] Lin Q Chiu APY Zhao S He D. Modeling the spread of Middle East respiratory syndrome coronavirus in Saudi Arabia. Stat Methods Med Res. (2018) 27:1968–78. 10.1177/0962280217746442 29846148

[33] National Health Commission of the Peoples's Republic of China. Clinical Diagnosis and Treatment Guidance of 2019 Novel Coronavirus (COVID-19) Caused Pneumonia. (2020). Available online at: http://www.nhc.gov.cn/yzygj/s7652m/202002/54e1ad5c2aac45c19eb541799bf637e9.shtml (cited July 21, 2020).

[34] Cruz CJP Ganly R Li Z Gietel-Basten S Gietel-Basten S. Exploring the young demographic profile of COVID-19 cases in Hong Kong: evidence from migration and travel history data. PLoS One. (2020) 15:e0235306. 10.1371/journal.pone.0235306 32589645 PMC7319322

[35] Roxby AC Greninger AL Hatfield KM Lynch JB Dellit TH James A. Detection of SARS-CoV-2 among residents and staff members of an independent and assisted living community for older adults - Seattle, Washington, 2020. MMWR Morb Mortal Weekly Rep. (2020) 69:416–8. 10.15585/mmwr.mm6914e2 32271726 PMC7147909

[36] Gudbjartsson DF Helgason A Jonsson H Magnusson OT Melsted P Norddahl GL. Spread of SARS-CoV-2 in the Icelandic Population. N Engl J Med. (2020) 382:2302–15. 10.1056/NEJMoa2006100 32289214 PMC7175425

[37] Tabata S Imai K Kawano S Ikeda M Kodama T Miyoshi K. Clinical characteristics of COVID-19 in 104 people with SARS-CoV-2 infection on the Diamond Princess cruise ship: a retrospective analysis. Lancet Infect Dis. (2020) 20, 1043–50. 10.1016/S1473-3099(20)30482-5 32539988 PMC7292609

[38] Lam HY Lam TS Wong CH Lam WH Leung CME Au KWA. The epidemiology of COVID-19 cases and the successful containment strategy in Hong Kong-January to May (2020). Int J Infect Dis. (2020) 98:51–8. 10.1016/j.ijid.2020.06.057 32579906 PMC7306206

[39] Day M. Covid-19: four fifths of cases are asymptomatic, China figures indicate. BMJ. (2020) 369:m1375. 10.1136/bmj.m1375 32241884

[40] Chen Y Xiong F Wang W Jiang K Ye X Deng G. The long persistence of pyrrolizidine alkaloid-derived pyrrole-protein adducts *in vivo*: kinetic study following multiple exposures of a pyrrolizidine alkaloid containing extract of Gynura japonica. Toxicol Lett. (2020) 323:41–7. 10.1016/j.toxlet.2020.01.021 31982501

